# Poly(Sulfobetaine Methacrylate-co-Vinyl Pyrrolidone) Hydrogels as Potential Contact Lenses Delivery Systems for Timolol Maleate

**DOI:** 10.3390/gels9020114

**Published:** 2023-01-30

**Authors:** Denitsa Nikolova, Christo Tzachev, Lachezar Christov, Elena Vassileva

**Affiliations:** 1 Laboratory on Structure and Properties of Polymers, Faculty of Chemistry and Pharmacy, University of Sofia, 1, James Bourchier Blvd., 1164 Sofia, Bulgaria; 2 Laboratory of Pharmaceutical Technology, Faculty of Chemistry and Pharmacy, University of Sofia, 1, James Bourchier Blvd., 1164 Sofia, Bulgaria; 3 Laboratory on Water Soluble Polymer, Polyelectrolytes and Biopolymers, Faculty of Chemistry and Pharmacy, University of Sofia, 1, James Bourchier Blvd., 1164 Sofia, Bulgaria

**Keywords:** poly(sulfobetaine methacrylate), poly(vinyl pyrrolidone), copolymers, contact lenses drug delivery, timolol maleate

## Abstract

The study reveals the development of novel hydrogels based on sulfobetaine methacrylate (SB) and vinyl pyrrolidone (VP) copolymers as potential contact lenses delivery systems of timolol maleate (TM). The novel copolymer networks demonstrated composition dependent swelling kinetics, where the hydrophilicity of VP and the physical network of SB monomeric units play significant roles. TM loading efficiency appeared to slightly depend on the copolymeric composition, increasing upon VP monomeric unit increase. In contrast, the TM release was prolonged when the SB monomeric units content in the copolymers increased, reaching full drug release for 48 h for the SB-rich networks. The transparency of the hydrogels was also studied and the obtained values demonstrate their applicability as potential materials for soft contact lenses. The study has revealed the potential of these novel copolymeric hydrogels as materials for contact lenses delivery systems of timolol maleate.

## 1. Introduction

Drug delivery via the ophthalmic pathway is a challenge that scientists have been trying to solve nowadays by developing advanced drug delivery systems. The widely used approach is based on eye drops, which are relatively cheap and easy to apply, although they have one very serious disadvantage—the very low drug bioavailability that they can ensure, which is estimated to be around 5% [1]. The remaining 95% of the drug is washed out of the eye, which increases the frequency of drug dosing and at the same time results into losing a vast amount of the active ingredient. One of the methods to keep the drug longer in the eye and thus to increase its bioavailability is by using drug-eluting contact lenses. The soft contact lenses are preferred for ophthalmic drug administration, as they ensure a better contact with the ocular surface and allow a greater flow of oxygen to the eye. Soft contact lenses are usually made of polymer hydrogels, the most common being poly(hydroxyethyl methacrylate) (pHEMA). pHEMA lenses have been studied as a carrier for active ingredients such as non-steroidal anti-inflammatory drugs [2], drugs for glaucoma treatment (e.g., timolol [3]), various antibiotics [4], antihistamines [5] and tear stimulants [6]. Although pHEMA has already proven its qualities for ophthalmic applications, the drug is released from pHEMA lenses very quickly, usually within an hour. That is why finding suitable materials that could control, e.g., slow down, the drug release from the contact lenses, could widen up the application of the contact lenses as drug dosage forms.

A recent literature search reveals a great interest towards advanced approaches for timolol maleate delivery. For example, Patel et al. [7] reported in situ hydrogel formulation for TM eye drops, in order to make them more viscous and hence to ensure that the higher drug amount will penetrate into the eye. Pasi et al. [8] developed mucoadhesive buccal hydrogel patches as a new approach for introducing timolol maleate in the body. Mirzaeei et al. [9] developed new TM sustained-released device based on polycaprolactone electrospun nanofibers. Our recent work [10] also focused on the developing of the novel delivery system, based on poly(sulfobetaine methacrylate) nanoparticles for prolonged TM delivery. Nevertheless, applying hydrogel contact lenses as a drug delivery system for TM is still in its infancy, which, however, reveals a promising potential to overcome the current obstacles in the field.

We focused our research on two polymers that are promising candidates to be used for contact lenses’ drug dosage forms manufacturing, namely poly(sulfobetaine methacrylate) (pSB) and poly(vinyl pyrrolidone) (pVP) (Table S1, Supplementary information (SI)). Both polymers are known to be highly biocompatible. pSB has biomimicking zwitterionic moieties in its side chains, which are known to impart the “stealth” effect, i.e., do not allow recognition by the host immune system, so it usually provokes a none to weak immune response. Moreover, pSB exhibits “smart” responsiveness to changes in temperature and salt concentration [11], which could allow a controlled triggering of the drug release upon changing the external conditions, e.g., upon application in the eye. pSB shows an upper critical solution temperature (UCST) type behavior in the range of 35–40 °C [12], which could be tuned via, e.g., copolymerization. As the overall average temperature of the human eye surface is *ca* 34.5 ± 0.8 °C (with minimum temperature of the cornea eye measured to be ~33.82 ± 1.10 °C and maximum temperature 35.41 ± 0.73 °C) [13], the ocular applications of pSB-based lenses would be expected to trigger the drug release. This trigger could be further enhanced by the pSB contact with tears, which have certain ionic strength and would provoke an additionally enhanced swelling of the pSB hydrogel. 

pVP is widely used in medicine and pharmacy for the manufacturing of drug carriers, implants, etc. pVP has a high oxygen permeability, and maintains an optimal water content and a high level of wetting, which are the main characteristics that a material for soft contact lenses should possess [14]. At the same time, the mechanical properties of pVP allow for making of contact lenses that fit well to the eyeball shape. Therefore, a hydrogel matrix based on these two polymers, pSB and pVP, would represent a promising ophthalmic drug delivery system with inherent biocompatibility.

Timolol maleate (TM) (Table S1, SI) is widely used nowadays for glaucoma treatment. It is a non-selective beta blocker, which reduces the intraocular pressure by blocking beta receptors in the ciliary body, thus reducing the intraocular fluid [15]. Patients who use TM must apply the TM eye drops several times a day, in order to keep its therapeutic dose. This not very patient-friendly manner of application is one of the reasons for seeking more advanced ways for TM ophthalmic applications. Thus, the development of novel TM carriers that are able to provide the therapeutic dose while avoiding a repeated daily intake of the medication as well as the waste of the active ingredient, is a challenge that seeks a solution. 

The aim of the study is to synthesize and characterize novel copolymeric hydrogels based on pSB and pVP as a way to create novel materials for contact lenses TM delivery systems. To this purpose, three different compositions of the copolymeric hydrogels were obtained, differing in their monomers’ molar ratios. The obtained hydrogels were characterized in terms of their swelling ability, TM loading efficiency and release profiles, as well as their transparency.

## 2. Results and Discussion

### 2.1. Equilibrium Swelling Ratio (ESR) of pSB-co-pVP Hydrogels

ESRs in distilled water of the three copolymeric hydrogels as well as the ESR of the pSB hydrogel are presented in Table 1. It is clearly observed that with decreasing the content of VP monomeric units in the copolymeric hydrogel, the ESR decreases. 

**Scheme 1:**
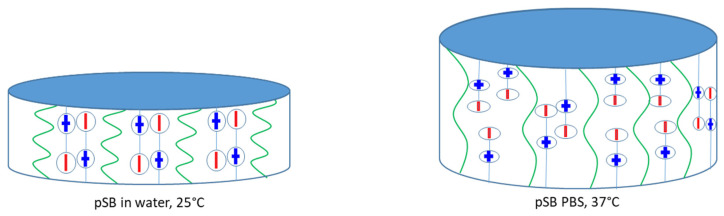
Dipole-dipole zwitterionic clusters acting as junctions of the pSB physical network.

This trend could be explained by two distinct features of the monomeric units, namely: (i) pVP is more hydrophilic as compared to pSB and (ii) pSB is able to form a physical network via dipole-dipole zwitterionic clusters (Scheme 1), which reduces its ESR. Thus, both factors define the observed decrease in ESR of the pVP-co-pSB hydrogels in distilled water, as the VP content decreases. 

It could be summarized that the proper choice of the copolymer’s components as well as the molar ratio between them allows for control on the swelling ability of the newly developed copolymeric hydrogels.

### 2.2. Elastic Modulus of pSB-co-pVP Hydrogels 

Elastic moduli of pSB-co-pVP hydrogels at their ESR in distilled water were determined following the Hertz contact theory (Table 2). The results demonstrate a slight increase in the elastic moduli as the SB content in the copolymeric hydrogels increases. This could be explained by the increase in the number of the dipole-dipole clusters (Scheme 1), which enhances the effect of the pSB physical network on the properties of the copolymeric networks. This result is in line with the ESR values presented in Table 1, where the same effect of the pSB physical networks on the swelling ability of the copolymeric networks is clearly observed.

### 2.3. Swelling Kinetics of pSB-co-pVP Networks

**Figure 1:**
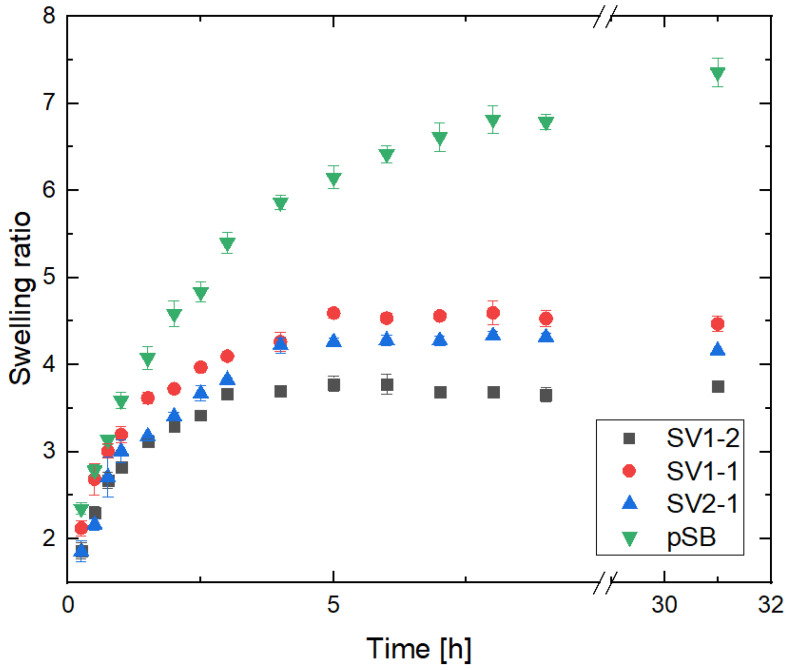
Swelling kinetics of pSB-co-pVP and pSB hydrogels in PBS at 37 °C.

The swelling kinetics of pVP-co-pSB hydrogels, under conditions that most closely resemble the in vitro drug release experiment, i.e., in phosphate buffer solution (PBS) at 37 °C, was studied (Figure 1). The swelling kinetics of the pSB network was also evaluated for the sake of comparison. The neat pSB has the highest swelling ratio as compared to the three copolymeric hydrogels (Figure 1), even though it has the lowest equilibrium swelling ratio in distilled water (Table 1). This can be explained by the existing physical network in pSB, formed via dipole-dipole clusters (Scheme 1). These clusters are stable in distilled water at room temperature but are destroyed (i) upon temperature increase, e.g., at 37 °C, which is the temperature used for the drug release as well as for the swelling kinetics studies; and (ii) upon salt addition, e.g., PBS is used in the drug release and the swelling kinetics studies, while the ESR data are obtained for swelling in distilled water. Thus, two of the conditions chosen for the swelling kinetics experiment destroy the pSB physical network, and the obtained hydrogel expands as compared to the pSB swelling in distilled water (Scheme 1). The same process of dipole-dipole clusters disruption lies also behind the upper critical solution temperature behavior of the neat pSB, i.e., the polymer expands upon temperature increase [16].

The copolymeric hydrogels show a less steep slope at the beginning, and lower swelling ratios at longer swelling times, as compared to the neat pSB. Similarly to pSB, their swelling ratios in PBS at 37 °C (Figure 1) are higher than in distilled water (Table 1). The reason is, again, the disruption of the physical network, defined by the presence of SB monomeric units, although in the copolymers the number of the physical network junctions is lower as compared to their number in the pSB, so the swelling conditions impact their SRs kinetics values less (Figure 1). Following this explanation, the observed lowest swelling ratio for the copolymeric hydrogel with the lowest SB monomeric units’ content, namely SV1-2, could have been expected. This is the sample where the number of the SB monomeric units is the lowest among all studied samples and thus the elevated temperature and the salt concentration influence its behavior less strongly as compared to the other pSB-co-pVP copolymers.

It is interesting to note that both other copolymeric hydrogels, namely SV1-1 and SV2-1, have very close swelling kinetics profiles, which means that both factors outlined above, namely the different hydrophilicity of both monomeric units as well as the pSB physical network, have comparable effects, but as they play against each other neither of them overwhelms the other in the SV1-1 sample. 

### 2.4. Differential Scanning Calorimetry (DSC) of pSB-co-pVP Networks

The thermal properties of the copolymeric networks were studied by DSC, and the thermograms (1st heating run) are presented in Figure S1, SI. The moisture (water amount) of each copolymeric network, stored under room conditions, was determined using Equation (2), and the results are presented in Table 3. In this way, the hygroscopicity, i.e., the ability of the copolymeric networks to absorb or adsorb moisture from the surrounding environment, was evaluated. 

The results demonstrated that the VP-rich copolymeric networks retain more moisture as compared to the pSB rich ones, which supports the role of the VP hydrophilicity in the overall behavior of the pSB-co-pVP networks. 

### 2.5. TM Entrapment Efficiency (EE) and Drug Loading Capacity (DLC) of pSB-co-pVP Hydrogels

**Figure 2:**
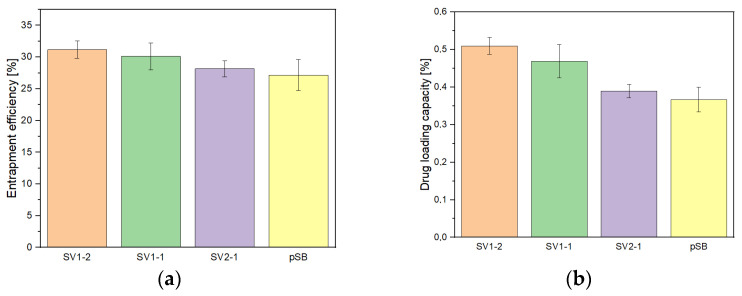
TM entrapment efficiency (EE) (**a**) and drug loading capacity (DLC) (**b**) for (pSB-co-pVP) hydrogels.

The TM entrapment efficiency (EE) for all copolymeric networks is presented in Figure 2a along with their drug loading capacity (DLC)(Figure 2b). The copolymers composition does not clearly influence the EE value, and all three copolymer networks have similar values of EE within the experimental error, which is ~30%,. The overall ANOVA and Tukey post hoc test results confirm this conclusion (see Tables S2 and S3, SI). 

The DLC, however, exhibits a clear dependence on the copolymer composition, as observed in Figure 2b: as the SB amount increases, the TM loading capacity increases, which is also confirmed by the overall ANOVA and Tukey post hoc test results (Tables S4 and S5, SI). The trend, observed for DLC in Figure 2b, could be explained either with (i) interactions between the VP monomeric units and TM, e.g., via hydrogen bonds and/or (ii) with enhanced swelling ability of the VP-rich copolymer networks, which also enhances the TM diffusion into them; i.e., purely diffusion-driven TM loading into the polymeric hydrogels takes place. The latter is in agreement with the ESR results presented in Table 1, which demonstrate the higher ability of the VP-enriched polymer networks to swell in distilled water (TM loading takes place in distilled water). Thus, the DLC dependence on the copolymeric composition could be explained by the enhanced swelling ability of the copolymeric network enriched in VP monomeric units, on the one hand, which allows more TM to enter the networks and thus to increase DLC, and the pSB physical network, on the other hand, which prevents the TM diffusion, as it defines higher crosslinking density of the SB-rich copolymeric networks. The currently widely applied timolol maleate eye drops are 0.125% solution of TM, applied two times per day. This ensures a daily dose of TM ~125 µg. As it was already mentioned above, the bioavailability of the TM delivered via eye drops is less than 5%, and usually around 1–2%, which means that the daily uptake of TM is around 2.5 µg [1]. On the other hand, it is believed that around 50% of the drug delivered via soft contact lenses reaches the cornea of the eye [2]. Applying these calculations for the pSB-co-pVP hydrogels as contact delivery system for TM shows that the drug loaded into the copolymeric hydrogels is around 300 µg, as the entrapment efficiency of around 30%. This makes a dose of 210 µg TM at 70% cumulative drug release, 105 µg TM of which is reaching the cornea. This illustrates well that a better performance could be expected from the developed within the study novel pSB-co-pVP hydrogels as TM delivery systems applied as contact lenses’ dosage form.

### 2.6. TM Release from pSB-co-pVP Hydrogels

**Figure 3:**
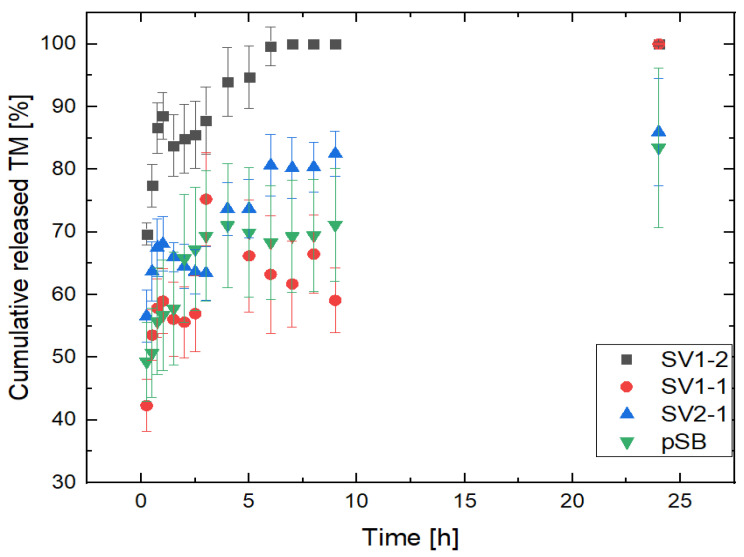
Release profiles of TM from the copolymeric hydrogels for 24 h.

The release profiles of TM from the copolymeric hydrogels are presented in Figure 3. All samples demonstrate a burst effect, as within the first 30 min more than 40% of the loaded TM is released. After the initial burst release, however, the TM release slows down and exhibits a clear dependence on the copolymers composition. The higher the SB monomeric units’ content is, the slower the TM release is observed (Figure 3). The time needed to attain full drug release (100%) also depends on the copolymeric composition: both samples with the highest SB content, namely SV2-1 and pSB, attain 100% of TM release for 48 h (Figure S2, SI). 

The sample with the highest VP content, SV1-2, on the other hand, is the fastest to release 100% of the drug (for ~6 h). This is not expected as the swelling kinetics curve for this sample shows the slowest swelling as well as the lowest swelling ratio among the three copolymeric hydrogels (Figure 1). Thus, if the drug release was governed only by the drug diffusion, defined by the polymer network swelling ability at the conditions used for the TM release experiment, SV1-2 should have been the slowest TM releasing hydrogel. This result could be considered as indirect indication that besides the network density that influences the drug diffusion, drug–polymer interactions also probably have a role for the TM release profile. The SV1-1 copolymeric hydrogel, where SB and VP monomeric units are 1:1, releases TM much slower after the initial burst—it releases around 60% of the drug within the first 9 h and 100% of the drug at the 24th hour (Figure 3). 

The copolymeric hydrogel with the highest SB content, SV2-1, releases TM faster than SV1-1, but slower than SV1-2 during the first 9 h. However, after the 9th hour, it releases the drug much slower and 100% of TM are released from this hydrogel after 48 h (Figure S2, SI). The TM release profile of the SV2-1 copolymeric hydrogel closely resemble the TM release from the neat pSB network (Figure 3). Both samples release the whole loaded TM amount for 48 h (Figure S2, SI) and the amount of TM released by them during the first 9 h is comparable. 

Since the error bars for the TM release profiles from pSB, SV1-1 and SV2-1 are too high, the ANOVA analysis was applied. The results demonstrate that there is a statistically significant difference between the TM release profiles obtained for the different polymer carriers. Moreover, ANOVA tests also demonstrated that the concentration of TM in the plateau regions is constant within the error bars (Figure S3 and Tables S6–S10, SI).

These results cannot be explained only by the swelling ability of the neat pSB and SV2-1 networks as under the same conditions used for the in vitro drug release the neat pSB swells much faster and reaches a much higher swelling ratio as compared to SV2-1 (Figure 1), while the swelling kinetics of SV2-1 is very close to the one of SV1-1, i.e., one could expect close TM release profiles for SV1-1 and SV2-1. This behavior could be related to the SB monomeric units in the polymer carrier, which could lead to either (i) interaction between the SB monomeric units and the drug TM or (ii) to the SB physical network formation, which imposes additional obstacles for the TM diffusion when the drug goes out of the polymeric carrier. This physical network is not a static, as it is gradually disrupted by the temperature and salt concentration (part of the experimental conditions for TM release experiment). Some time is needed in order for the SP physical network to be disrupted and to release more and more TM, which extends the TM releasing time to 48 in case of the polymeric carriers with the highest SB content. Thus, Figure 3 illustrates the influence of the SB monomeric units on the TM release profiles well.

### 2.7. Kinetic Study on TM Release

TM release profiles were further studied by applying the main mathematical models that describe the drug release kinetics (Table 4). Neither from the four listed models fully describes the TM release profiles in Figure 3, although the Higuchi model has the highest correlation coefficient for all four studied samples. This result is somehow expected, as the Higuchi diffusion model is developed exactly for the hydrogel drug release systems. 

The Korsmeyer-Peppas model also has high correlation coefficients, especially for the SV2-1 and pSB samples; however, it cannot be applied for SV1-2, where the burst effect is too large (almost 70% of the drug was released within the first 15 min). The diffusion exponents (n) for the studied hydrogels are less than 0.45, which indicates a pseudo-Fickian diffusion mechanism of the TM release. This means that the diffusion of the drug timolol maleate within the hydrogels is much faster than the hydrogels’ relaxation [16]. This conclusion is in line with the observed SB content influence on the TM release. The imposed SB physical network “hardens” the copolymer network and strongly entraps the drug inside. The more “hardened” the polymer network, the bigger will be the difference between the rates of the TM diffusion and the polymer chains’ relaxation. As the in vitro release conditions (temperature and PBS) start to disrupt the SB clusters, the drug is liberated gradually and starts to leave the hydrogel, and this is the way the SB content strongly influences the TM release.

### 2.8. Attenuated Total Teflectance Infrared Spectroscopy (ATR-IR)

**Figure 4:**
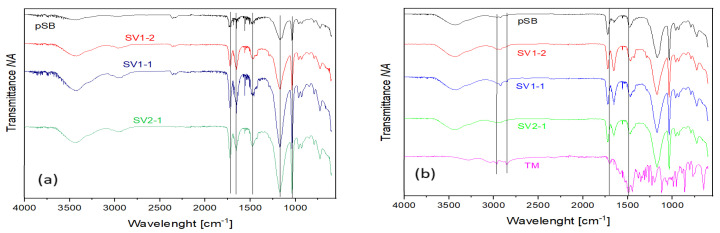
FTIR spectra of (**a**) non-drug-loaded and (**b**) TM-loaded pSB-co-pVP and pSB networks.

In order to check whether the drug TM interacts with the polymer carriers, ATR-IR was used (Figure 4). First, we studied the neat polymeric carriers, namely, the three copolymeric networks as well as the neat pSB (Figure 4a). Two broad bands are observed for all samples at high wavelengths. The broad band that appears at ~3500 cm^−1^ is due to –O-H stretching in the SB and VP monomeric units, while the broad band between 2700 and 3100 cm^−1^ is attributed to the symmetric and asymmetric stretching of CH_2_ groups from the pyrrole ring in VP as well as from the SB backbone. 

In the neat pSB spectrum, the band at 1720 cm^−1^ is attributed to the C=O stretching vibrations from the SB monomeric units, whose band is also observed in the spectra of all copolymeric networks. The band at 1650 cm^−1^ is attributed to the C-N^+^ stretching in the SB monomeric unit, which in the neat pSB spectrum is small and narrow. In the copolymeric networks, however, this band is more intensive, due to overlapping with the C=O stretching of VP. Normally, the band for the C=O stretching in VP appears around 1660–1670 cm^−1^; but, when shifted below 1664 cm^−1^, it is a common indication for strong hydrogen bonds formation. Such hydrogen bonds could be formed between VP monomeric units and water molecules absorbed in the networks upon room conditions’ storage (see Table 3). Another evidence for the hydrogen bonds formation is the increase in the broadness and intensity of the O-H stretching band at 3500 cm^−1^ [17]. The band observed at 1470 cm^−1^ is due to the quaternary ammonium group in SB. The most intensive bands that appear at 1170 cm^−1^ and 1035 cm^−1^ are due to the S=O stretching and vibration in SB [18,19,20]. 

The IR spectra of TM-loaded polymer networks as well as of the TM itself are presented in Figure 4b. The bands in the neat TM spectrum could be assigned as follows: the characteristic bands that correspond to the O–H vibrations are observed at 2965 cm^−1^ and at 2856 cm^−1^, the former being shifted to 2920 cm^−1^ in the TM-loaded hydrogels’ spectra, which indicates an interaction between the drug and the polymeric carriers. The other two characteristics for TM bands at 1700 cm^−1^ and 1490 cm^−1^ are due to the N–H stretching and vibrations [16, 17]. No clear shift in the bands for the NH group in TM when TM is loaded into polymer networks are observed; thus, the IR spectra cannot unequivocally prove the hydrogen bonds’ formation between the polymer and the drug. 

DSC analysis was also used to study the drug–polymer carriers’ interaction. To this purpose, Tg values of both the non-drug-loaded and TM-loaded networks were determined (Table S11, SI) using the respective thermograms (Figures S1, S4 and S5, SI). However, no clear dependence of the Tg values on the copolymers’ composition was observed. Thus, our attempts to register any interaction between the drug TM and the copolymeric networks were not successful.

The moisture of room conditioned copolymeric networks, loaded with TM, has also been evaluated; however, no clear dependence on the copolymeric composition (Table 3) is observed. It should be noted here that the TM-loaded samples demonstrate higher moisture as compared to the non-drug-loaded ones for most copolymeric samples. The neat pSB is exception from this observation; thus, it could be concluded that indeed the VP monomeric units are with higher hydrophilicity as compared to the SB ones and impart higher moisture in the VP-rich copolymer networks. 

### 2.9. Light Transmittance of TM-Loaded pSB-co-pVP Hydrogels

**Figure 5:**
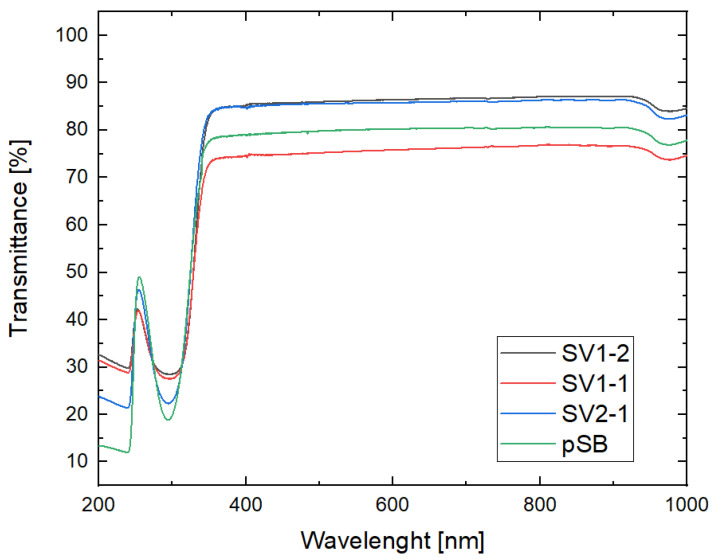
Transmittance spectra of TM-loaded hydrogels.

The aimed application of the pSB-co-pVP hydrogels as drug delivering contact lenses requires that they are transparent and do not blur the patients’ vision. That is why the light transmittance of the TM-loaded copolymeric hydrogels was determined (Figure 5). 

The absorbance of TM, loaded in the respective hydrogels, is clearly observed having an absorption maximum at 294 nm. The results in Figure 5 demonstrate that in the visible part of the spectrum, the SV1-1 sample possesses the lowest transparency, around 70%. The pSB hydrogel, loaded with TM, has a transparency around 75%, while samples SV1-2 and SV2-1 have higher light transmittance, which is approximately 85%. Transparency of 85% is sufficient to ensure clear vision through the materials when used to produce contact lenses. Gulsen et al. have reported the transmittance of the soft contact lens, made of pHEMA, to be 87% [21], while Zhu et al. [22] claim that transmittance higher than 67% does not affect the optical properties of the materials. Thus, SV1-2 and SV2-1 are the candidates with the best transparency, although the other two hydrogels are also viable materials for contact lenses’ production.

We have also estimated the transmittance of the polymeric hydrogels after 5 h TM release in order to check whether the release process will affect their transparency. The results, shown in Figure S6, SI, demonstrate that the transmittance of all pSB-co-pVP hydrogels increases after 5 h TM release, most probably due to the decrease in the drug content rather than to a further networks expansion. It should be reminded here that the release experiment started from the swollen state of the hydrogels; thus, the hydrogels do not expand significantly during the release experiment itself. The drug release kinetics (Figure 3) shows that the sample with the highest TM content at 5 h release time (pSB) has the lowest transmittance in Figure S6, although it has the highest swelling degree at the same time as compared to the three copolymeric networks (Figure 1), i.e., it should have the highest swelling ratio at these conditions. On the other hand, the hydrogel, which after 5 h has released ~95% of the loaded drug, has the highest transparency, namely SV1-2 has 95% transmittance (Figure S6). Thus, the transmittance of the copolymeric hydrogels increases after TM release for all copolymeric hydrogels but is the same for the pSB network. Thus, one cannot expect that the TM release will worsen neither the hydrogels’ transmittance nor the wearing comfort—we did not detect an increase in the thickness of the hydrogels after 5 h release time. 

**Figure 6:**
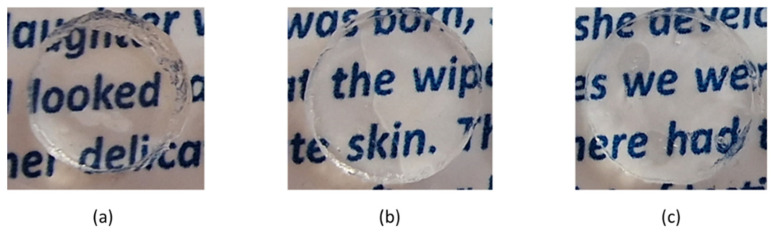
Photographic images of TM-loaded (**a**) SV1-2, (**b**) SV1-1 and (**c**) SV2-1 hydrogels.

Further evidence of the transparency of the copolymeric hydrogels are the photographs of these materials presented in Figure 6. These photos clearly show that the TM-loaded hydrogels are completely transparent when loaded with TM and before starting its release. For the sake of comparison, the photo of the TM-loaded pSB hydrogel is presented in Figure S7, SI.

All hydrogels appeared not to transmit much light in the UV-B region (Figure 5), e.g., their transmittance is between 19 and 28%, depending on their composition, which means that they block between 72 to 81% of the UV-B radiation (Table 5). 

Recent studies have outlined the relation between the UV-B radiation present in sunlight, and the formation of natural lens cortical opacities. Moreover, as UV-B rays have shorter wavelength, but higher energy level as compared to UV-A, they do not penetrate much in the tissues. Thus, they hit the corneal epithelium, and are known to accelerate the loss of corneal epithelial cells and enhance the related photo keratitis [23]. That is why soft contact lenses are required to also have UV protection, which is usually imparted by adding special additives. TM-loaded pSB-co-pVP hydrogels exhibit an inherent UV-B blocking ability, which is an additional advantage for their application as soft contact lenses.

## 3. Conclusions

Copolymer hydrogels, based on sulfobetaine methacrylate, vinyl pyrrolidone and poly(ethylene glycol) diacrylate, were successfully synthesized for the first time. Three different copolymers’ composition, as well as neat pSB hydrogel, were studied as potential materials for making soft contact lenses to be used in ocular timolol maleate delivery. The copolymers’ composition appeared to influence the drug loading capacity, namely the copolymers rich in VP monomeric units showed higher DLC as compared to SB-rich ones. The copolymer’s composition also influenced the TM release profiles, and the increase in the SB monomeric units resulted in slower TM release. The kinetic models applied to the TM release profiles demonstrate that they all exhibit pseudo-Fickian diffusion for the TM release. The presence of the SB-based physical network implies additional constraints and slows down the TM release, the effect being dependent on the SB content in the polymeric carrier. The results from the study reveal that SB/VP-based copolymeric networks are appropriate as materials for contact lenses’ TM delivery, and the copolymers’ composition could be used to prolong the TM release profiles.

## 4. Materials and Methods

### 4.1. Materials

Monomers N-(3-sulfopropyl)-N-(methacroyloxyethyl)-N,N-dimethylammonium betaine (sulfobetaine, SB), and 1-vinyl-2-pyrrolidone (vinyl pyrrolidone, VP), the crosslinking agent poly(ethylene glycol) diacrylate (PEGDA, Mn~575), initiator potassium persulfate (K_2_S_2_O_8_), potassium chloride (KCl), and potassium phosphate monobasic (KH_2_PO_4_) used for phosphate buffer saline solution (PBS) preparation, were purchased by Merk, Darmstadt, Germany. The other salts used for PBS preparation, sodium chloride (NaCl), and sodium phosphate dibasic (Na_2_HPO_4_), were purchased, respectively, by Fluka, Seelze, Germany, and Zedelgem, Belgium. The drug timolol maleate was kindly provided by Antibiotic-Razgrad AD, Razgrad, Bulgaria. All reagents were used as received, without further purification. 

### 4.2. Methods

#### 4.2.1. Preparation of pSB-co-pVP Hydrogels and Neat pSB 

A total of 1 M aqueous solution of SB was prepared, and a defined amount from the second monomer, VP, was added in order to keep molar ratios between both monomers SB:VP to be 1:2, 1:1 or 2:1, respectively (Table 6). The initiator K_2_S_2_O_8_, with a concentration of 0.1 mol%, relative to the total number of moles of both monomers, was added. The crosslinking agent PEGDA, whose concentration was 2 mol%, relative to the total number of moles of both monomers, was also added. The reaction mixture was stirred for 10 min at room temperature. Then, it was placed between two reaction plates separated by a spacer with defined thickness. The crosslinking polymerization was carried out at 60 °C for 6 h. The obtained hydrogels were immersed in distilled water to remove the residuals. The water was changed daily until it was clear from residuals as proved by UV spectroscopy. 

For the sake of comparison, the neat poly(sulfobetaine methacrylate) (pSB) network was synthesized following the procedure described above. Briefly, 1 M aqueous solution of the SB monomer was prepared. The initiator K_2_S_2_O_8_ and the crosslinking agent PEGDA were added in concentrations 0.1 mol% and 2 mol% in relation to the number of SB moles, respectively. The reaction mixture was stirred for 10 min at room temperature; then, it was placed between two glass plates separated by a rubber separator with defined thickness, and the crosslinking polymerization was carried out at 60 °C for 6 h. The obtained pSB network was purified in the same way as described above.

#### 4.2.2. Equilibrium Swelling Ratio (ESR)

The ESRs of the neat pSB and pSB-co-pVP hydrogels were determined gravimetrically. To this purpose, at least three pieces from each copolymer composition network were immersed into distilled water and their weight was measured daily until reaching a constant value. Then, ESR of the hydrogels was calculated using the equation:

(1) $$\mathrm{ESR}=\frac{m_{w}-{\;m}_{d}}{m_{d}}$$

where m_w_ is the weight of the swollen pieces at their equilibrium swelling and m_d_ is their initial weight when dry.

#### 4.2.3. Elastic Modulus of pSB-co-pVP Hydrogels

The elastic moduli of pSB-co-pVP hydrogels, as well as of the neat pSB network, were determined using the Hertz contact theory [24]. Briefly, a metal ball with a radius R_ball_ was used as a load, and was placed on the hydrogel surface. This leads to the hydrogel surface deformation. The depth of the obtained indentation (D) was measured by a cathetometer and following the Equations (2) and (3), the elastic modulus (EM) of the hydrogel was calculated:

(2) $$R_{\mathrm{contact}}=\sqrt{{(2R}_{\mathrm{ball}\;}\times \;D)\;-{\;D}^{2}}$$

(3) $$\mathrm{EM}=\left(\frac{9}{16}\right)\;\times \;\frac{R_{\mathrm{ball}}{\;\times \;w}_{\mathrm{ball}}\;\times \;g}{R_{\mathrm{contact}}^{3}}$$

where R_contact_ is the radius of the contact area between the ball and the hydrogel, w_ball_ is the weight of the metal load and g is the acceleration of gravity.

#### 4.2.4. Swelling Kinetics

The swelling kinetics of pSB-co-pVP hydrogels was studied under conditions resembling the drug release study. To this purpose, three disk-shaped pieces from each copolymeric network were immersed in 50 mL PBS with pH = 7.4 and placed at 37 ± 0.5 °C. At certain time intervals, the samples were taken out, wiped of the excess water, and weighed in order to determine their swelling ratio using Formula (1), where w_d_ is their weight at the defined swelling time. 

#### 4.2.5. Timolol Maleate (TM) Loading in pSB-co-pVP Networks

The TM loading of the obtained pSB-co-pVP networks was performed by the immersion of at least three dry disk-shaped pieces from each copolymeric composition in 0.5 mL aqueous solution of TM with concentration 1 mg/mL for at least 24 h. The samples were used to study the TM release immediately after they were taken out from the drug solution.

#### 4.2.6. Attenuated Total Reflectance Infrared Spectroscopy (ATR-IR)

Dry neat (non-drug-loaded) and TM-loaded pSB-co-pVP networks were studied by using ATR-IR spectroscopy. The regime of the attenuated total reflectance of the IRAffinity-1 Shimadzu Fourier Transform Infrared spectrophotometer with the MIRacle Attenuated Total Reflectance Attachment, Kyoto, Japan was used. 

#### 4.2.7. Differential Scanning Calorimetry (DSC)

The thermal properties of room conditionsdried neat and TM-loaded pSB-co-pVP networkswere studied by using Q200 TA Instruments, New Castle, NCL, USA. The samples were heated from −90 °C to 220 °C, then cooled to −90 °C, and heated again to 220 °C using 10 °C per minute heating rate under nitrogen flow of 50 mL/min.

Using the 1st heating run, the water content (moisture) of these samples, stored under room conditions, was determined using the equation:

(4) $$W_{c}=\frac{\Delta H}{\Delta H_{H_{2}O}}\;\times \;100$$

where, W_c_ is the water content (in %), ∆H is the enthalpy of the endothermic peak from the 1st heating run, related to the evaporation of the water present in the room-conditioned sample, and ∆H_H2O_ is the enthalpy of the evaporation of 100% of the water taken to be 2400 J/g according to [25]. The DSC measurements were run for three independent samples for two samples, namely SV1-1 and SV2-1, and the error for the moisture determination for these samples was evaluated to be 3.7% for SV1-1 and 9.7% for SV2-1.

#### 4.2.8. Light Transmittance 

In view of the planned application as soft contact lenses, the light transmittance of the newly developed hydrogels was evaluated. For this experiment, UV-Vis spectrophotometer (JASCO V-730 Spectrophotometer, Heckmondwike, UK) was used. The TM-loaded hydrogels were placed on the outer surface of the quartz cuvette, and it was placed in the spectrophotometer. Then, the transmittance was measured in the wavelength region between 200 and 1000 nm. 

#### 4.2.9. TM Entrapment Efficiency (EE) and Drug Loading Capacity (DLC)

**Figure 7:**
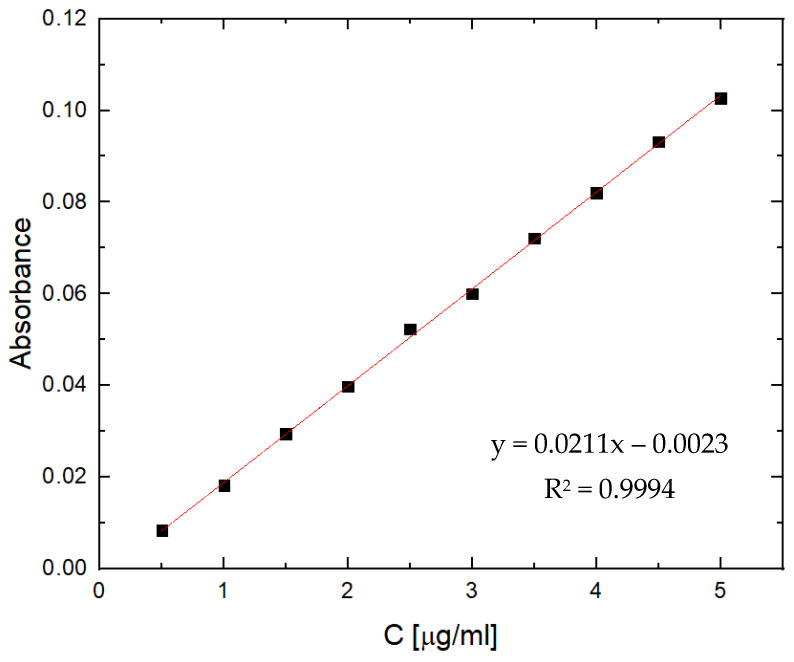
Calibration curve for timolol maleate at 294 nm.

To determine TM entrapment efficiency, at least 5 dry disk-shaped pieces from each copolymeric composition were immersed in the 0.5 mL TM solution with concentration 1 mg/mL for 24 h. The absorbance of the solutions, left after the hydrogels were taken out, was measured with the UV-Vis spectrophotometer at 294 nm, as this is the absorption maximum determined for TM (Figure S8, SI). In order to quantify the measured absorbance, a calibration curve was obtained (Figure 7) using TM solutions in PBS with known concentrations. 

The following equation was used to estimate the EE of TM:

(5) $$\mathrm{EE}=\frac{m_{\mathrm{total}}-{\;m}_{\mathrm{res}}}{m_{\mathrm{total}}}\;\times \;100$$

where m_total_ is the amount of TM that was dissolved in the initial TM solution used for the drug loading of each sample, and m_res_ is the amount of the non-loaded TM, detected in the solution left after the loading procedure.

The drug loading capacity (DLC) was also evaluated using the following equation

(6) $$\mathrm{DLC}=\frac{m_{\mathrm{total}}-{\;m}_{\mathrm{res}}}{m_{\mathrm{carrier}}}\;\times \;100$$

where m_carrier_ is the weight of the carrier before drug loading.

#### 4.2.10. TM Release Profiles

Copolymeric hydrogels, freshly removed from the TM solution where the drug loading took place, were placed in 50 mL of freshly prepared PBS solution, with pH 7.4. The experiment was set at 37 °C ± 0.5, mimicking the ocular conditions. At fixed time intervals, an aliquot was taken, and its absorbance was measured with the UV spectrophotometer; then, it was returned to the solution where the in vitro experiment took place to keep its volume constant.

#### 4.2.11. Kinetic Study of the TM in vitro Dissolution

The main kinetic models for drug dissolution were applied for the obtained TM release profiles, in order to study the drug release kinetics in more detail. These are described by the following equations: 

Zero order [26]:

(7) $$M_{t}{=M}_{0\;}{-\;K}_{0}$$

First order [26]:

(8) $$M_{t}{=M}_{0}e^{K_{1}t}$$

Higuchi’s diffusion model [27]

(9) $$M_{t}=\mathrm{KH}\sqrt{t}$$

Korsmeyer–Peppas model [28]:

(10) $$\frac{M_{t}}{M_{\infty }}{=K}_{\mathrm{KP}}t^{n}$$

where M_t_ is the amount of drug released at time t, M_0_ is the initial amount of the drug loaded in the drug carrier, and K_0_, K_1_, K_H_ and K_KP_ are kinetic constants.

## Figures and Tables

**Table 1:** Equilibrium swelling ratios of pVP-co-pSB hydrogels in distilled water at room temperature.

Sample Designation	ESR
SV1-2	2.55 ± 0.02
SV1-1	2.2 ± 0.03
SV2-1	1.98 ± 0.02
pSB	1.72 ± 0.04

**Table 2:** Elastic moduli of pVP-co-pSB hydrogels at their ESR in distilled water at room temperature.

Sample Designation	Elastic Modulus [MPa]
SV1-2	8.82 ± 0.04
SV1-1	8.78 ± 0.09
SV2-1	9.07 ± 0.05
pSB	9.22 ± 0.04

**Table 3:** Water content, Wc, in room-conditioned pSB-co-pVP networks, both non-drug-loaded and TM-loaded.

Sample	Wc of Non-Loaded Samples [%]	Wc of TM-Loaded Samples [%]
SV1-2	29.5%	38.6%
SV1-1	27.8%	42.3%
SV2-1	25.0%	24.5%
pSB	27.1%	20.2%

**Table 4:** Kinetic models describing the TM release profile from pSB-co-pVP hydrogels.

Model/Sample		SV1-2	SV1-1	SV2-1	pSB
Zero order	K_0_	1.023	1.826	1.163	1.227
	R_0_^2^	0.775	0.739	0.619	0.644
First order	K_1_	0.005	0.011	0.0069	0.008
	R_1_^2^	0.408	0.662	0.584	0.574
Higuchi model	K_H_	6.721	9.268	8.974	7.321
	R_H_^2^	0.678	0.691	0.812	0.829
Korsmeyer-Peppas	K_KP_	-	54.475	70.958	56.079
	n	-	0.104	0.162	0.099
	R_KP_^2^	-	0.551	0.998	0.913

**Table 5:** Optical transmittance of pSB-co-pVP and pSB hydrogels, loaded with TM.

Sample	UV-B (280–315 nm)	UV-A (316–380 nm)	SWB * (381–460 nm)	LWB ** (461–500 nm)
SV1-2	28.55%	81.43%	85.61%	85.84%
SV1-1	27.53%	72.12%	74.78%	75.06%
SV2-1	22.48%	82.58%	85.16%	85.5%
pSB	19.16%	77.12%	79.22%	79.70%

* SWB-short-wavelength blue region, ** LWB-long-wavelength blue region.

**Table 6:** Description of pSB-co-pVP networks.

Sample Designation	SB (Molar Parts)	VP (Molar Parts)
SV1-2	1	2
SV1-1	1	1
SV2-1	2	1
pSB	1	-

* Concentrations of the initiator K_2_S_2_O_8_ (0.1 mol%) and the crosslinking agent PEGDA (2 mol%) were kept constant in relation to the total monomer concentration for all samples.

## Data Availability

The raw/processed data required to reproduce these finfings cannot be shared at this time as the data also form part of an ongoing study.

## Supplementary Materials

The following supporting information can be downloaded at: https://www.mdpi.com/article/10.3390/gels9020114/s1, Table S1. Chemical formulas of the reagents used within the study along with their role. Figure S1. DSC thermograms (1st heating run) for neat and TM loaded copolymeric networks as well as for the neat and TM loaded pSB network. Table S2. Overall ANOVA analysis of the entrapment efficiency (EE) data, presented in Figure 2a. Table S3. Tukey post hoc test for the entrapment efficiency (EE) data, presented in Figure 2a. Table S4. Overall ANOVA analysis of the drug loading capacity (DLC) data, presented in Figure 2b. Table S5. Tukey post hoc test for the drug loading capacity (DLC) data, presented in Figure 2b. Figure S2. Release profiles of TM from the copolymeric hydrogels for 48 h. Figure S3. Release profiles of TM from hydrogels in the range from the 30 min to the 5th hour, where a plateau was observed. Table S6. ANOVA analysis for both points from the TM release curve obtained for SV1-2 sample, which are designated in black ellipses in Figure S3. Table S7. ANOVA analysis for both points from the TM release curve obtained for SV1-1 sample, which are designated in red ellipses in Figure S3. Table S8. ANOVA analysis for both points from the TM release curve obtained for SV2-1 sample, which are designated in blue ellipses in Figure S3. Table S9. ANOVA analysis for both points from the TM release curve obtained for pSB sample, which are designated in green ellipses in Figure S3. Table S10. Tukey post hoc test for the marked points in Figure S3. Table S11. Glass transition temperatures of non-drug-loaded and TM-loaded pSB-co-pVP networks (determined from Figure S1). Figure S4. DSC thermograms (2nd heating run) for neat copolymeric networks as well as for the neat pSB network. Figure S5. DSC thermograms (2nd heating run) for TM loaded copolymeric networks as well as for TM loaded pSB network. Figure S6. Transmittance spectra of TM loaded hydrogels after 5 h drug release at 37 °C in PBS. Figure S7. Photographic image of TM-loaded pSB hydrogel. Figure S8. UV spectra of timolol maleate aqueous solutions, used for the preparation of the calibration curve. All spectra have maximum at 294 nm.
