# Supplementary material for: Poly(Sulfobetaine Methacrylate-co-Vinyl Pyrrolidone) Hydrogels as Potential Contact Lenses Delivery Systems for Timolol Maleate
Source: Gels. 2023 Jan 30;9(2):114. doi: 10.3390/gels9020114 (PMC9956182; doi:10.3390/gels9020114)
Supplement: Supplementary file 1 [file gels-09-00114-s001.zip › gels-2149546-supplementary.pdf]

Supplementary Information

Table S1. Chemical formulas of the reagents used within the study along with their role.

Name of the reagent	Role of the reagent	Chemical formula
[2-(Methacryloyloxy)ethyl]dimethyl-(3-sulfopropyl)ammonium hydroxide<br>Sulfobetaine methacrylate	Monomer	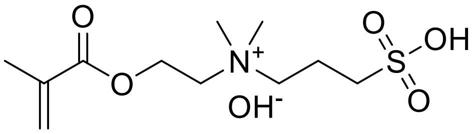
N-Vinyl pyrrolidone	Monomer	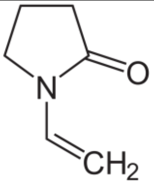
Potassium persulfate	Initiator	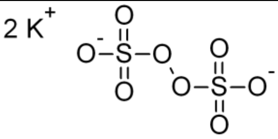
Poly(ethylene glycol diacrylate)	Crosslinking agent	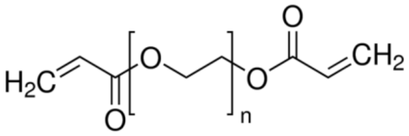
Timolol maleate	Drug for glaucoma therapy	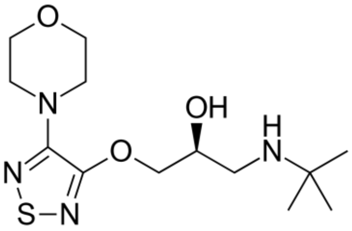

Thermal properties of (pSB-co-pVP) networks as studied by DSC

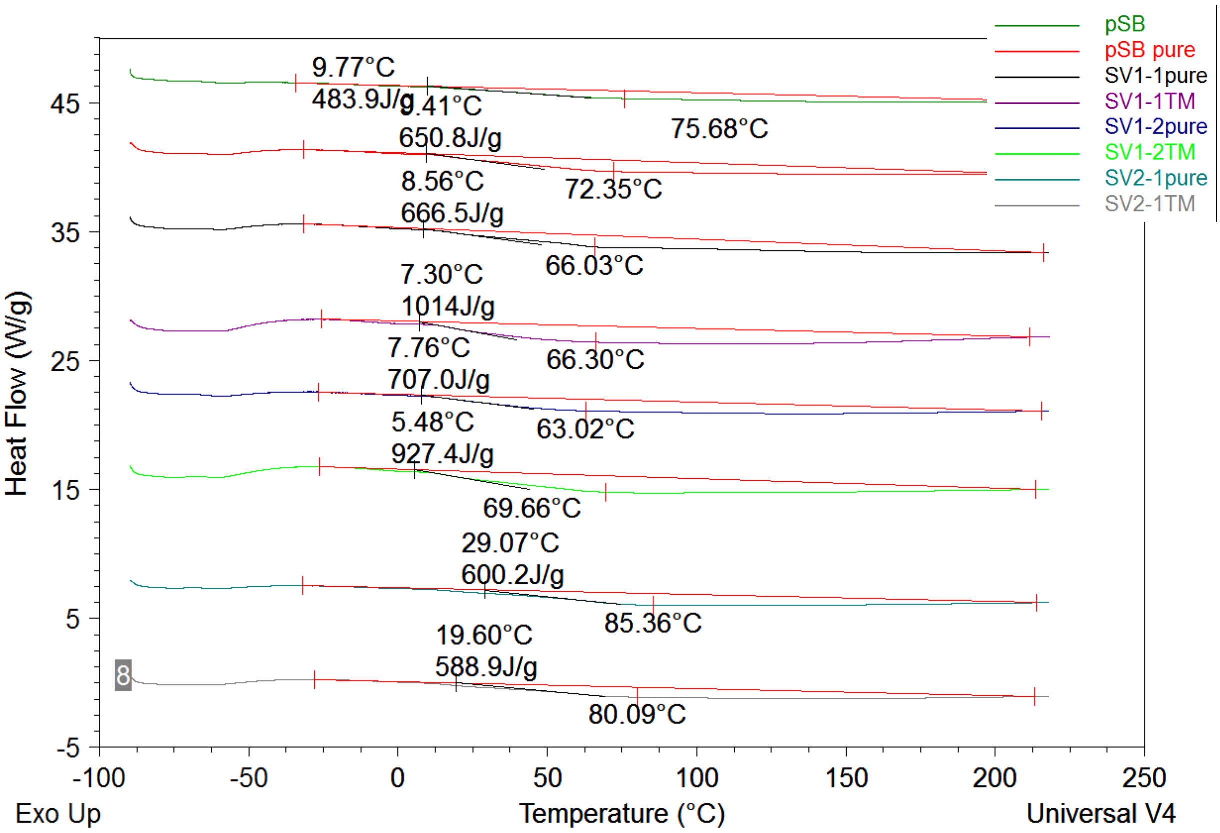

Figure S1. DSC thermograms (1st heating run) for neat and TM loaded copolymeric networks as well as for the neat and TM loaded pSB network.

Overall ANOVA analysis for TM entrapment efficiency (EE) in pSB-co-pVP hydrogels

One-way ANOVA and Tukey Tukey post hoc test were performed using OriginPro 2018 software (OriginLab Corporation, www.originlab.com).

The overall ANOVA analysis was applied for the entrapment efficiency data (Figure 2a) and the results are presented in Table S2.

Table S2. Overall ANOVA analysis of the entrapment efficiency (EE) data, presented in Figure 2a.

	DF	Sum of Squares	Mean Square	F Value	Prob>F
Model	3	79,76437	26,58812	0,92032	0,44177
Error	33	953,37472	28,89014		
Total	36	1033,13908			

At the 0.05 level, the population means are **not** significantly different.

Table S3. Tukey post hoc test for the entrapment efficiency (EE) data, presented in Figure 2a.

	MeanDiff	SEM	q Value	Prob	Alpha	Sig*	LCL	UCL
SV1-1 SV1-2	-1,05113	2,29189	0,6486	0,96746	0,05	0	-7,25057	5,14831
SV2-1 SV1-2	-2,99977	2,34849	1,80641	0,58344	0,05	0	-9,35231	3,35276
SV2-1 SV1-1	-1,94864	2,34849	1,17344	0,83996	0,05	0	-8,30118	4,40389
pSB SV1-2	-4,00203	2,89904	1,95228	0,52012	0,05	0	-11,84377	3,83971
pSB SV1-1	-2,9509	2,89904	1,43951	0,74023	0,05	0	-10,79264	4,89084
pSB SV2-1	-1,00226	2,94398	0,48146	0,98618	0,05	0	-8,96558	6,96107

*Sig equals 1 indicates that the difference of the means is significant at the 0.05 level. Sig equals 0 indicates that the difference of the means is not significant at the 0.05 level.

Overall ANOVA analysis for drug loading capacity (DLC) of pSB-co-pVP hydrogels

The overall ANOVA analysis was applied for the DLC data (Figure 2b) and the results are presented in Table S4.

Table S4. Overall ANOVA analysis of the drug loading capacity (DLC) data, presented in Figure 2b.

	DF	Sum of Squares	Mean Square	F Value	Prob>F
Model	3	0,11715	0,03905	4,04443	0,01461
Error	34	0,32828	0,00966		
Total	37	0,44544			

At the 0.05 level, the population means are significantly different.

Table S5. Tukey post hoc test for the drug loading capacity (DLC) data, presented in Figure 2b.

	MeanDiff	SEM	q Value	Prob	Alpha	Sig*	LCL	UCL
SV1-1 SV1-2	-0,04071	0,0419	1,37424	0,76627	0,05	0	-0,15388	0,07245
SV2-1 SV1-2	-0,12035	0,0419	4,06204	0,03357	0,05	1	-0,23351	-0,00719
SV2-1 SV1-1	-0,07963	0,0419	2,6878	0,24684	0,05	0	-0,19279	0,03353
pSB SV1-2	-0,1431	0,053	3,81861	0,05007	0,05	0	-0,28624	3,37446E-5
pSB SV1-1	-0,10239	0,053	2,73218	0,23407	0,05	0	-0,24553	0,04075
pSB SV2-1	-0,02276	0,053	0,60729	0,97302	0,05	0	-0,1659	0,12038

*Sig equals 1 indicates that the difference of the means is significant at the 0.05 level. Sig equals 0 indicates that the difference of the means is not significant at the 0.05 level.

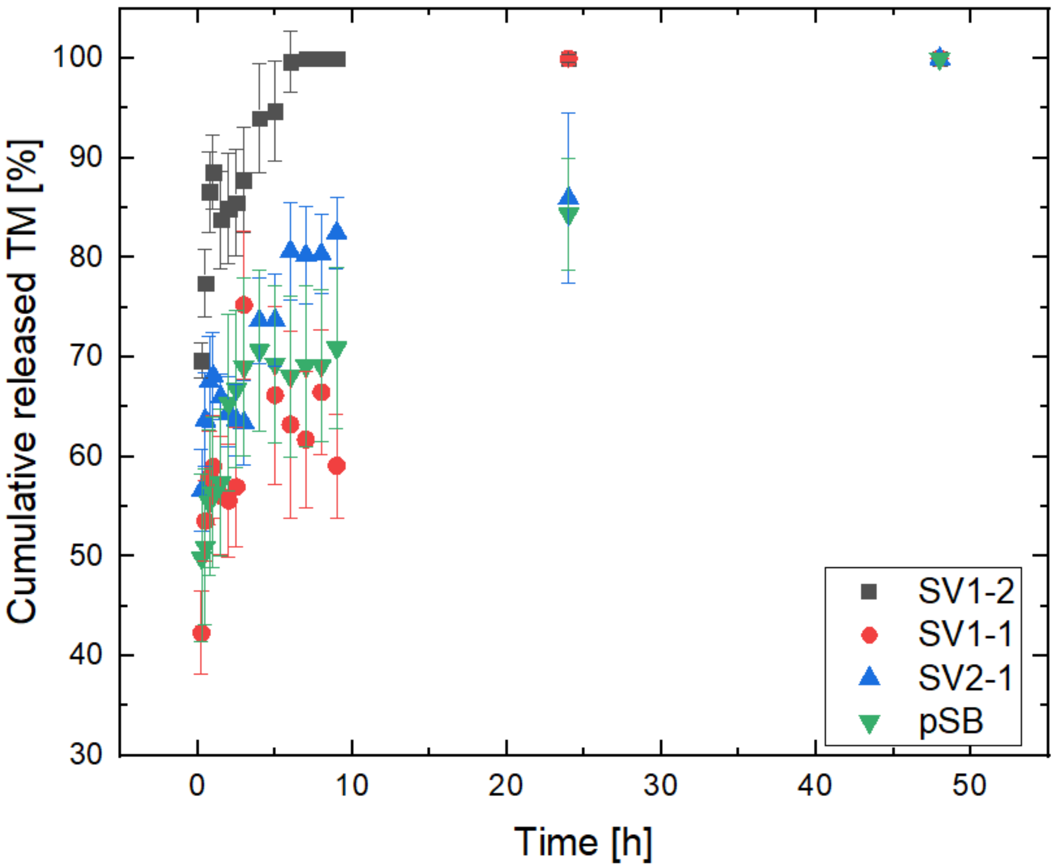

Figure S2. Release profiles of TM from the copolymeric hydrogels for 48 h.

Statistical analysis for TM release from pSB-co-pVP hydrogels

One-way ANOVA and Tukey test were performed using OriginPro 2018 software (OriginLab Corporation, www.originlab.com).

The ANOVA tests were applied for two points from each TM release curve, obtained for the different copolymeric hydrogels, which points differed most significantly in their cumulative released TM. The points are designated in Figure S4 by ellipses, using the colour which was used for the entire respective curve.

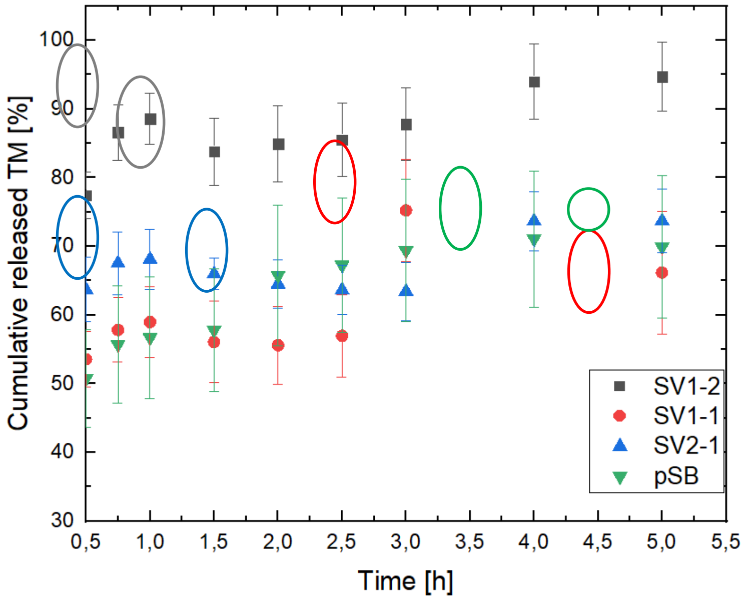

Figure S3. Release profiles of TM from hydrogels in the range from the 30 minutes to the 5th hour, where a plateau was observed.

Table S6. ANOVA analysis for both points from the TM release curve obtained for SV1-2 sample, which are designated in black ellipses in Figure S4.

	DF	Sum of Squares	Mean Square	F Value	Prob>F
Model	1	33,90301	33,90301	0,59625	0,4831
Error	4	227,44078	56,8602		
Total	5	261,34379			

The direct comparison of the points where possible decrease in the drug release is detected for SV1-2 sample, using ANOVA test, indicates that no statistically significant difference between both points is detected. That means that within the experimental error these two points from the TM release curve for SV1-2 sample are similar and no real decrease exists.

Table S7. ANOVA analysis for both points from the TM release curve obtained for SV1-1 sample, which are designated in red ellipses in Figure S4.

	DF	Sum of Squares	Mean Square	F Value	Prob>F
Model	1	123,21959	123,21959	0,61215	0,47771
Error	4	805,16478	201,29119		
Total	5	928,38437			

The direct comparison of the points where possible decrease in the drug release is detected for the TM release plateau obtained for SV1-1 sample, using ANOVA test, indicates that no statistically significant difference between both points is detected. That means that within the experimental error these two points from the TM release curve for SV1-1 sample are similar and no real decrease exists.

Table S8. ANOVA analysis for both points from the TM release curve obtained for SV2-1 sample, which are designated in blue ellipses in Figure S4.

	DF	Sum of Squares	Mean Square	F Value	Prob>F
Model	1	32,93858	32,93858	0,58538	0,48686
Error	4	225,07561	56,2689		
Total	5	258,01419			

The direct comparison of the points where possible decrease in the drug release is detected in the TM release profile from SV2-2 sample, using ANOVA test, indicates that no statistically significant difference between both points is detected. That means that within the experimental error these two points from the TM release curve for SV2-1 sample are similar.

We have also analyzed the plateau observed for the TM release from the neat pSB and the results are presented in Table S6.

Table S9. ANOVA analysis for both points from the TM release curve obtained for pSB sample, which are designated in green ellipses in Figure S4.

	DF	Sum of Squares	Mean Square	F Value	Prob>F
Model	1	2,02747	2,02747	0,0066	0,93916
Error	4	1228,93632	307,23408		
Total	5	1230,96379			

The performed ANOVA indicates that a decrease in the amount of drug released was not actually observed also for the TM release from the neat pSB hydrogel.

Tukey post hoc test also confirmed that there is no significant difference between each two analyzed points, at 0.05 significance level.

Table S10. Tukey post hoc test.

	MeanDiff	SEM	q Value	Prob	Alpha	Sig*	LCL	UCL
SV1-2	-4,75416	6,15685	1,09202	0,4831	0,05	0	-21,84813	12,33982
SV1-1	-9,06346	11,58422	1,10648	0,47771	0,05	0	-41,22607	23,09914
SV2-1	-4,68605	6,12475	1,08201	0,48686	0,05	0	-21,69091	12,31881
pSB	-1,1626	14,31163	0,11488	0,93916	0,05	0	-40,89763	38,57242

*Sig equals 1 indicates that the difference in the means is significant at the 0.05 level. Sig equals 0 indicates that the difference of the means is not significant at the 0.05 level.

Differential scanning calorimetry (DSC) of TM-loaded pSB-co-pVP networks

Table S11. Glass transition temperatures of non-drug-loaded and TM-loaded pSB-co-pVP networks (determined from Figure S1).

Sample	Tg of non-loaded samples [°C]	Tg of loaded samples [°C]
SV1-2	18.67 °C	17.54 °C
SV1-1	17.23 °C	17.75 °C
SV2-1	18.26 °C	16.87 °C
pSB	16.66 °C	18.05 °C

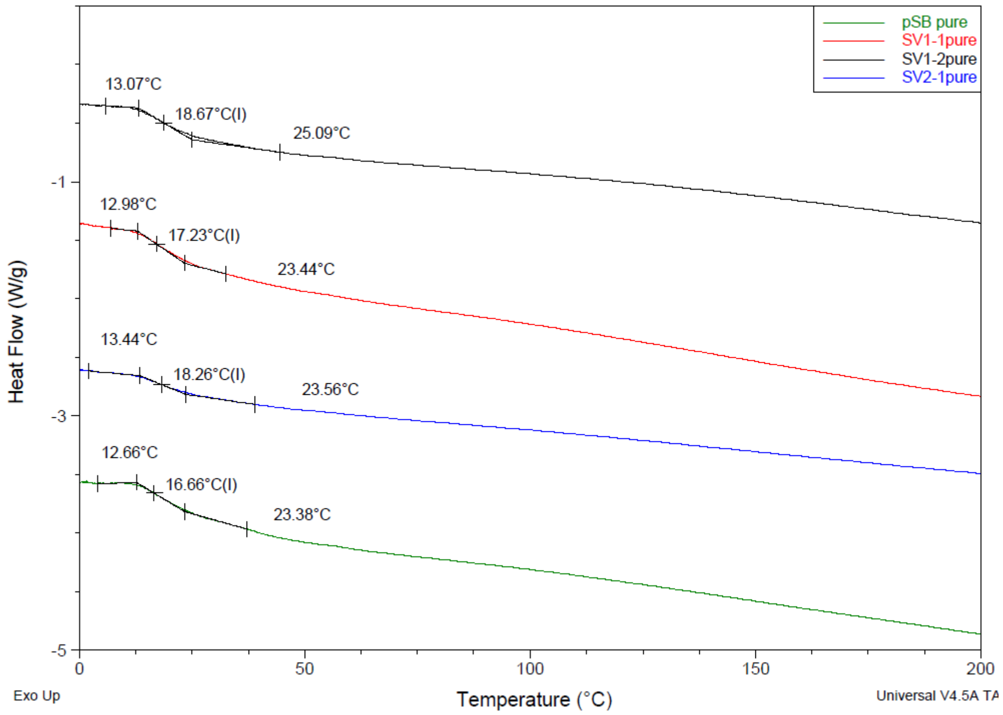

Figure S4. DSC thermograms (2nd heating run) for neat copolymeric networks as well as for the neat pSB network.

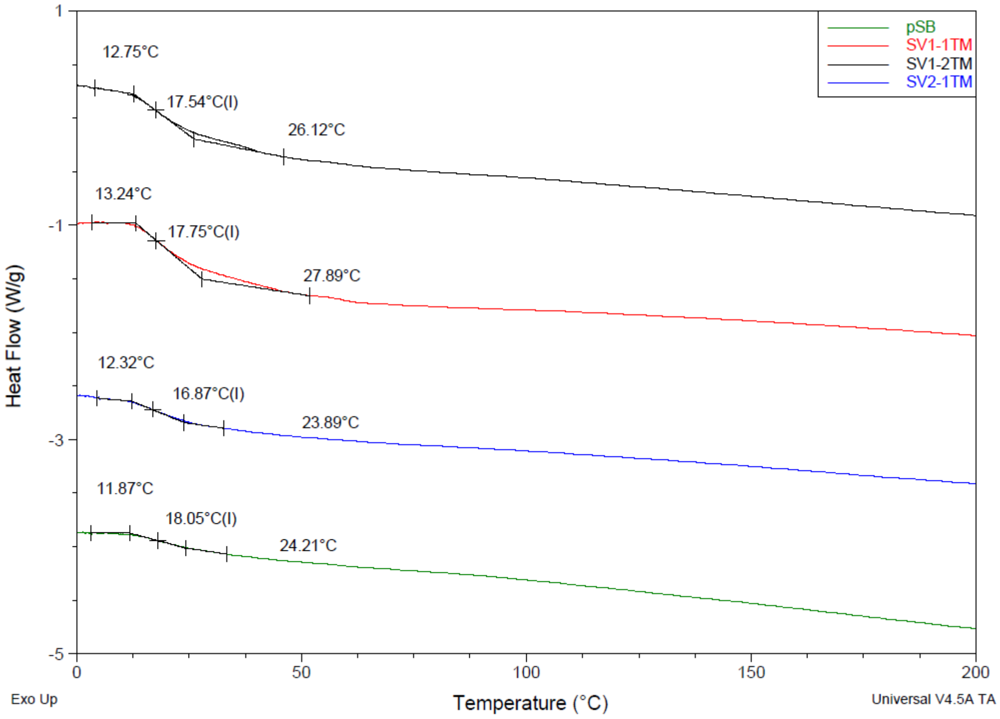

Figure S5. DSC thermograms (2nd heating run) for TM loaded copolymeric networks as well as for TM loaded pSB network.

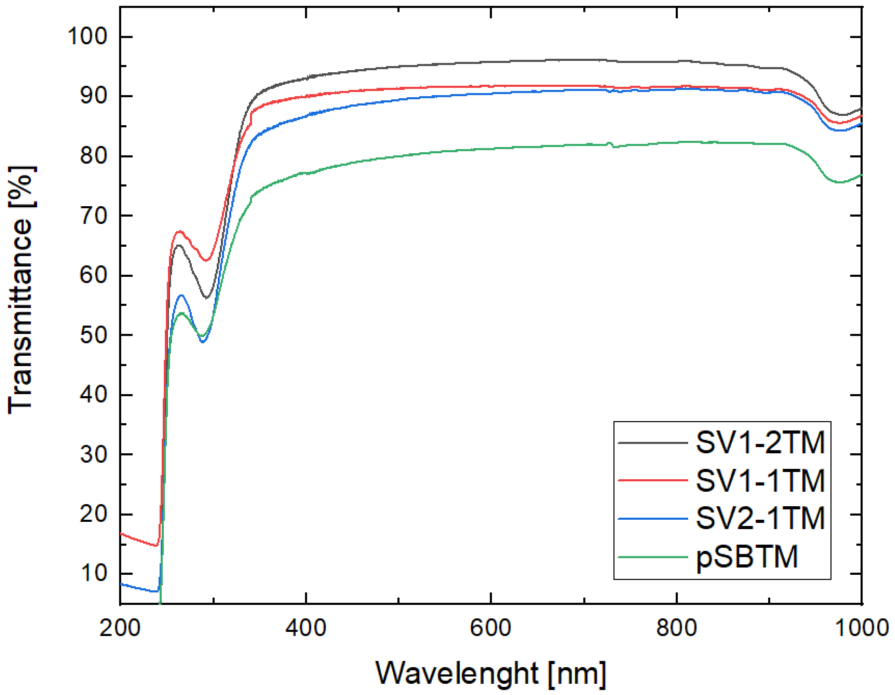

Figure S6. Transmittance spectra of TM loaded hydrogels after 5h drug release at 37 °C in PBS.

Light transmittance of TM-loaded pSB hydrogel

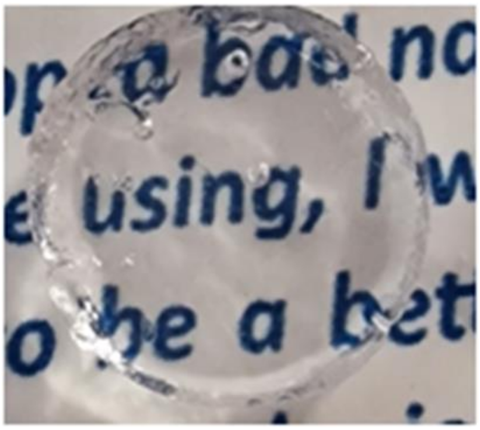

Figure S7. Photographic image of TM-loaded pSB hydrogel.

The bubbles appearance in the neat pSB could be avoided by a step of degassing, e.g. via purging nitrogen flow, which is also the approach used for soft contact lenses production from other polymers, e.g. silicon based.

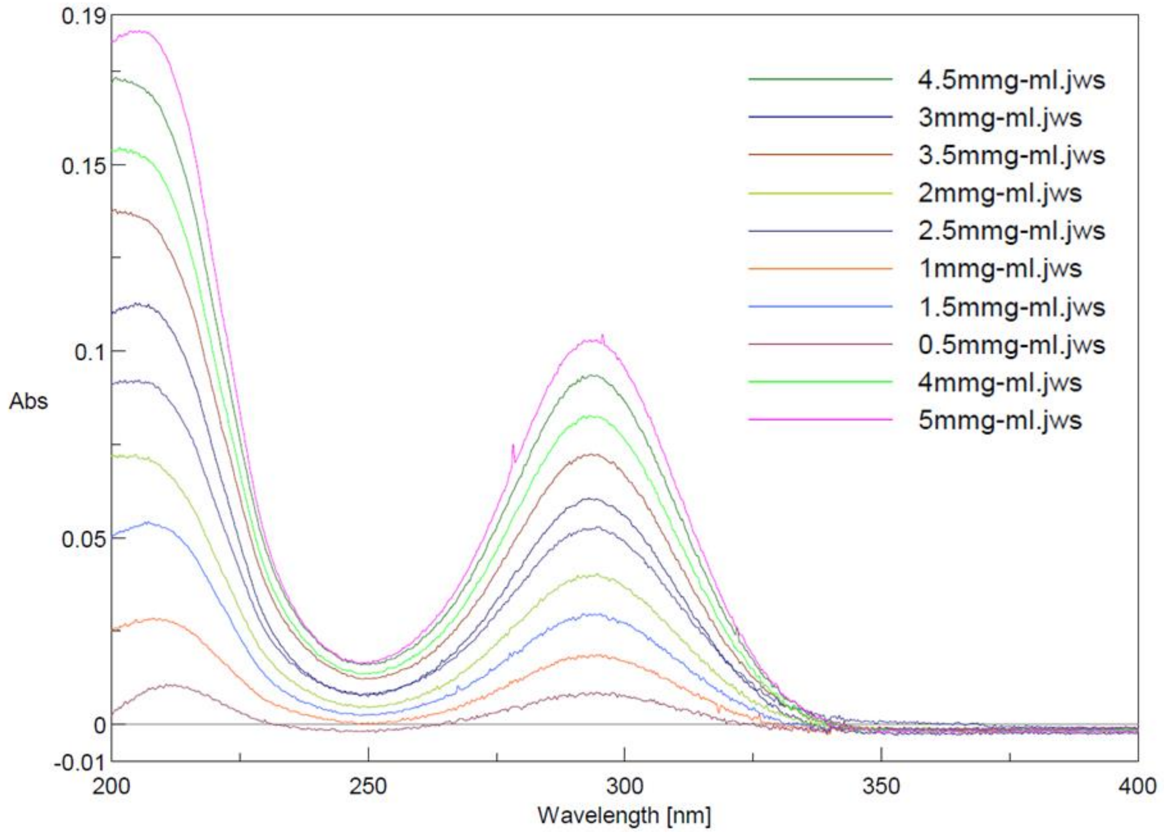

Figure S8. UV spectra of timolol maleate aqueous solutions, used for the preparation of the calibration curve. All spectra have maximum at 294 nm.
